# The Isolation of *Aeromonas* Species and Other Common Enteric Bacterial Pathogens from Patients with Gastroenteritis in an Australian Population

**DOI:** 10.3390/microorganisms9071440

**Published:** 2021-07-03

**Authors:** Christopher Yuwono, Michael C. Wehrhahn, Fang Liu, Stephen M. Riordan, Li Zhang

**Affiliations:** 1 School of Biotechnology and Biomolecular Sciences, University of New South Wales, Sydney, NSW 2052, Australia; c.yuwono@student.unsw.edu.au (C.Y.); fang.liu@unsw.edu.au (F.L.); 2 Douglass Hanly Moir Pathology, 14 Giffnock Ave, Macquarie Park, NSW 2113, Australia; 3 Gastrointestinal and Liver Unit, Prince of Wales Hospital, University of New South Wales, Sydney, NSW 2031, Australia; Stephen.Riordan@sesiahs.health.nsw.gov.au

**Keywords:** *Aeromonas*, *Aeromonas veronii*, *Campylobacter*, *Salmonella*, gastroenteritis

## Abstract

*Aeromonas* species are emerging human enteric pathogens. However, systematic analysis of *Aeromonas* species infection in human gastroenteritis in comparison with other enteric bacterial pathogens in the Australian population is lacking. Here we analysed the isolation of *Aeromonas* species and other bacterial pathogens in five consecutive years (2015–2019) from 375,842 stool samples of patients with gastroenteritis in a large Australian diagnostic laboratory and identified a subset (48 isolates) of *Aeromonas* isolates to species level, using multilocus phylogenetic analysis. *Aeromonas* species were the third most common bacterial pathogens, following *Campylobacter* and *Salmonella* species. *Aeromonas* infection rate was significantly correlated with increasing age (*p* < 0.001). *Aeromonas* species were more often isolated in warm seasons and in males than females (*p* < 0.001). Five *Aeromonas* species were identified. Most of the infections were from three species, namely *Aeromonas veronii* (52%), *Aeromonas caviae* (27%) and *Aeromonas hydrophila* (12.5%). The majority of patients with *Aeromonas* species infection did not have a documented overseas travel history. The findings from this study support the importance of *Aeromonas* species in human gastroenteritis and suggest that the sources of *Aeromonas* infection in Australian patients should be further investigated.

## 1. Introduction

Members of the *Aeromonas* genus, which currently contains 36 species, are Gram-negative rod-shaped motile or non-motile facultative anaerobes [1]. *Aeromonas* species cause diseases in both humans and animals, such as fish and reptiles. A majority of *Aeromonas* infections in humans are caused by five *Aeromonas* species, namely *Aeromonas caviae*, *Aeromonas veronii, Aeromonas dhakensis*, *Aeromonas hydrophila* and *Aeromonas media* [1].

*Aeromonas* species cause a range of human diseases. The extra-intestinal diseases caused by *Aeromonas* species in humans most commonly consist of wound infection and bacteraemia [1,2,3,4,5,6,7]. *Aeromonas* species are also emerging human enteric pathogens, causing various gastrointestinal disorders, such as gastroenteritis [7,8,9,10,11,12,13], traveller’s diarrhoea [12] and biliary tract infections [14,15,16]. *Aeromonas veronii* was also found to be more prevalent in patients with inflammatory bowel disease [17].

A variety of virulence factors in *Aeromonas* species, such as flagella, pili, capsules, enterotoxins, extracellular degradative enzymes and haemolysins, have been reported [18,19,20,21]. A Shiga-like toxin was also detected in *Aeromonas* species isolated from patients with gastroenteritis [22,23].

Despite the increasing importance of *Aeromonas* species in causing human gastroenteritis, systematic analysis of their infection in human gastroenteritis in comparison with other human enteric bacterial pathogens in Australian population is lacking. In this study, we analysed the isolation of *Aeromonas* species and other enteric bacterial pathogens in five consecutive years, from 2015 to 2019, using data from a large private Australian diagnostic laboratory serving predominantly community-based patients. A subset of *Aeromonas* isolates was also identified at the species level.

## 2. Materials and Methods

### 2.1. Clinical Data of Enteric Bacterial Pathogen Isolation

The data analysed in this retrospective study were provided by the diagnostic laboratory Douglass Hanly Moir Pathology. The laboratory is based in Sydney, Australia, receiving specimens from the state of New South Wales. The data analysed in this study were isolations of bacterial pathogens from stool samples of patients with gastroenteritis in this laboratory during 2015–2019. The identification of enteric bacterial pathogens was based on the standard protocols in this laboratory. The analysis focussed on *Aeromonas* species, with an additional five enteric pathogens also analysed for comparison. The isolation of *Aeromonas* species was conducted by following the procedures for isolation of enteric bacterial pathogens at the Douglass Hanly Moir Pathology. Briefly, the presumptive *Aeromonas* isolates were collected from either xylose lysine deoxycholate (XLD) agar plates (pale yellow or yellow pink flat colonies), thiosulfate citrate bile-salts sucrose (TCBS) agar plates (yellow or green flat colonies) or horse blood agar (HBA) plates containing an ampicillin AMP25 disc (Oxoid™, Scoresby, VIC, Australia). The presumptive *Aeromonas* isolates collected from XLD and HBA plates were subjected to an oxidase test. The oxidase-positive isolates from XLD and HBA plates, as well as isolates from TCBS plates, were then further subjected to matrix assisted laser desorption ionization/time of flight mass spectrometry (MALDI/TOF-MS) (Vitek MS, BioMerieux, North Ryde, NSW, Australia) for *Aeromonas* identity confirmation. Confirmed isolates were reported as “*Aeromonas* species” given the potential for misidentification of *Aeromonas* beyond the genus level by MALDI/TOF-MS. Other enteric bacterial pathogens were identified either to the genus or species level.

### 2.2. Statistical Analyses

A logistic regression analysis was performed by using a binomial generalised linear model to assess the associations between patient age and gender with the isolation rates of different bacterial pathogens. The odds ratio, the 95% confidence interval and the *p*-value were generated by the model. For this part of the analysis, 287 individuals whose age and gender identities were not disclosed in the information provided to the diagnostic laboratory were excluded. Spearman’s rank correlation coefficient was used to examine the correlations between the isolation rates of different bacterial pathogens with the local monthly mean maximum temperatures from January 2015 to December 2019 that were obtained from the Australian Government Bureau of Meteorology website. The statistical analyses were performed by using R software (v. 4.0.4), along with RStudio (v. 1.4.1106), and *p* < 0.05 indicates a statistical significance.

### 2.3. Identification of Aeromonas Species

The identification of *Aeromonas* species was conducted for 48 randomly selected *Aeromonas* isolates obtained during 2019, using multilocus phylogenetic analysis (MLPA) of seven housekeeping genes, namely *gyr*B, *rpo*D, *gyr*A, *rec*A, *dna*J, *dna*X and *atp*D, as previously described [24]. We sequenced the genomes of these 48 *Aeromonas* isolates for a different study, the sequences of seven genes were extracted from the sequenced bacterial genomes and used in this study for *Aeromonas* species identification. The sequences of these seven housekeeping genes of known 32 *Aeromonas* species (three strains from each species were used, if available) were obtained from the NCBI genome database. The genomes of the remaining four species, namely *Aeromonas enterica*, *Aeromonas crassostreae*, *Aeromonas aquatilis* and *Aeromonas intestinalis*, were not available on NCBI, and the reported identification of these four species was only based on the partial sequences of six housekeeping genes; therefore, they were excluded for analysis. Phylogenetic trees were generated by the neighbour-joining and maximum likelihood methods, as previously described, using the MEGA X program [25,26,27]. The sequences of the seven housekeeping genes of the 48 *Aeromonas* species analysed in this study were submitted to GenBank under accession numbers MW837843–MW837890, MW837891–MW837938, MW837939–MW837986, MW837987–MW838034, MW838035–MW838082, MW838083–MW838130 and MW838131–MW838178.

### 2.4. Ethics Approval

This is a retrospective study analysing data obtained from a diagnostic laboratory that did not involve patient consent. This project has been approved by the University of New South Wales HREAP Executive (HC200755).

## 3. Results

### 3.1. Aeromonas Species Were the Third Most Common Bacterial Pathogens Isolated from Stool Samples of Patients with Gastroenteritis Following Campylobacter and Salmonella Species

A total of 375,842 stool samples processed for detection of enteric bacterial pathogens, using bacterial cultivation, at the Douglass Hanly Moir Pathology laboratory, during 2015–2019, were analysed. The isolated bacterial enteric pathogens during 2015–2019 are shown in Table 1; these pathogens were reported in this diagnostic laboratory at either genus or species level. *Aeromonas* species were the third most common enteric bacterial pathogens, with the isolation rate (positive isolations in 10,000 samples) being 56.73. *Campylobacter* species were the most common enteric bacterial pathogens, and the isolation rate was 308.56. *Salmonella* species were the second most common enteric bacterial pathogens, with the isolation rate being 138.09. The isolation rates of *Shigella* species, *Plesiomonas shigelloides* and *Vibrio* species were 6.84, 6.07 and 2.89, respectively. Various other enteric bacterial pathogens were also isolated, but with low isolation rates; their data were therefore not further analysed (Table 1).

### 3.2. Aeromonas Species Were the Sole Enteric Bacterial Pathogens in Most of the Positive Cases

*Aeromonas* species were the sole enteric bacterial pathogens in 82% (1755/2132) of stool samples that were positive for *Aeromonas* species isolation (Table 2). In the remaining 18% (377/2132) of samples, *Aeromonas* species were co-isolated with other human enteric pathogens (Table 2).

### 3.3. Travel History of Patients with Positive Aeromonas Species Isolation

Of the patents who were positive for *Aeromonas* species isolation in the five years between 2015 and 2019, 4.08% of them had “travel” or “overseas” listed as part of the clinical history supplied. These were 3.97% and 7.03% for patients who were positive for isolation of *Campylobacter* and *Salmonella* species, respectively. For patients who were positive for *P. shigelloides*, *Shigella* and *Vibrio* species, 23.68%, 20.54% and 21.3% had travel history, respectively.

### 3.4. Isolations of Aeromonas Species and Additional Five Enteric Bacterial Pathogens in Patients of Different Age Groups and Gender

**Figure 1:**
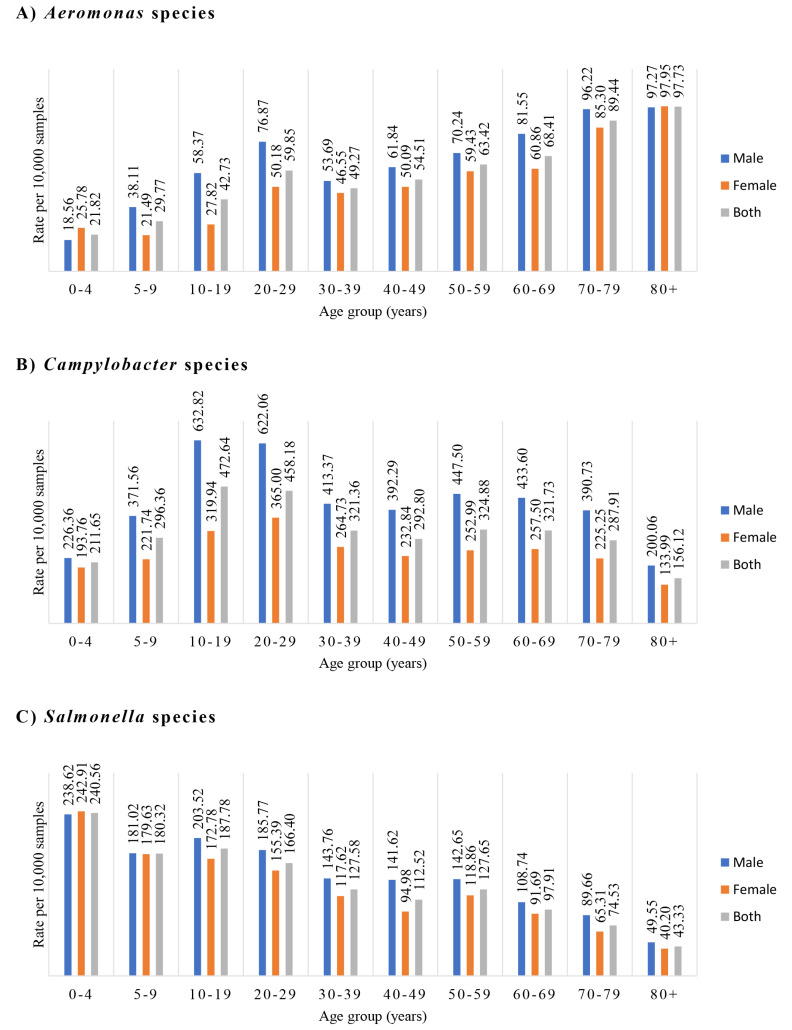
Isolation of *Aeromonas species*, *Campylobacter* species and *Salmonella* species during 2015–2019 from different age groups and gender. The bacterial pathogens were isolated from faecal samples of patients with gastroenteritis. The isolation rates were the positive isolations per 10,000 samples. (**A**) *Aeromonas* species, (**B**) *Campylobacter* species and (**C**) *Salmonella* species. *Aeromonas* species in human gastroenteritis showed a novel two peaks (20–29 age group and over 80 age group) infection pattern. The isolation of *Aeromonas* species was positively associated with age (*p* < 0.001) and significantly higher in male patients than female patients (*p* < 0.001). *Campylobacter* species was more isolated in the 10–29 age group; the isolation rate was negatively associated with patient age (*p* < 0.001) and was significantly higher in male patients than female patients (*p* < 0.001). *Salmonella* species was most often isolated in children of 0–4 age group; the isolation rate was negatively associated with patient age (*p* < 0.001) and was significantly higher in male patients than female patients (*p* < 0.001).

The *Aeromonas* species isolate rates during the five years between 2015 and 2019 are shown in Figure 1A. The *Aeromonas* species isolation rate was low in young children of 0–4 years old (21.82/10,000). It then gradually increased as the children got older, and it reached a peak in the age group of 20–29 years old, with the isolation rate being 59.85. *Aeromonas* species isolation rates in individuals of the 30–39-years-old and 40–59-years-old groups then decreased, with the isolation rates in these two age groups being 49.27 and 54.51, respectively. Interestingly, the *Aeromonas* species isolation started to increase again in the age group of 50–59 years old (isolation rate being 63.42), which continued to increase and reached a second peak in individuals over 80 years old (isolation rate being 97.73). There was a significant association between *Aeromonas* isolation rate and the increase of patient age; the *p*-value was < 0.001, and the odds ratio was 1.015 (95% confidence interval, 1.013–1.016). Male patients had a significantly higher isolation rate than female patients (0.6% vs. 0.5%); the *p*-value was < 0.001, and the odds ratio was 1.204 (95% confidence interval, 1.104–1.312).

For *Campylobacters* species isolation, the 10–19- and 20–29-years-old groups had the highest isolate rates, being 472.64 and 458.18, respectively (Figure 1B). There was a significant association between *Campylobacter* species isolation rate and the decrease of patient age; the *p*-value was < 0.001, and the odds ratio was 0.998 (95% confidence interval, 0.998–0.999). Male patients had a significant higher *Campylobacter* isolation rate than female patients (3.97% vs. 2.47%, *p* < 0.001); the odds ratio was 1.617 (95% confidence interval, 1.558–1.679).

For *Salmonella* species isolation, young children (0–4 years old) had the highest isolation rate, being 240.56. The isolation rate gradually decreased as the age increased, with individuals over 80 years old having the lowest isolation rate, being 43.33 (Figure 1C). There was a significant association between *Salmonella* species isolation rate and decreasing age, the *p*-value was < 0.001 and the odds ratio was 0.985 (95% confidence interval, 0.984–0.986). The isolation rates in male and female patients were significantly different (1.59% vs. 1.24%), the *p*-value was < 0.001 and the odds ratio was 1.154 (95% confidence interval, 1.092–1.219).

The positive isolations of *Aeromonas, Campylobacter* and *Salmonella* species in individual years, from 2015 to 2019, in different age groups are shown in Table 3.

**Figure 2:**
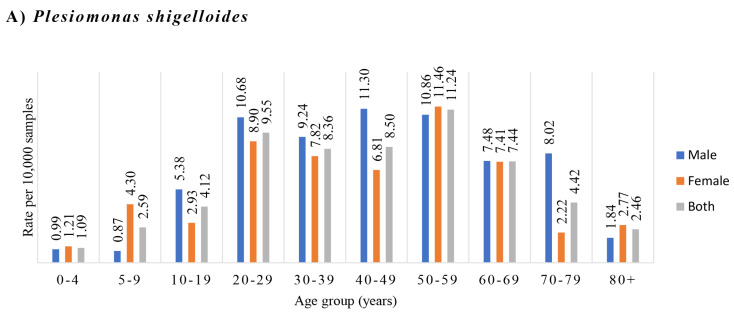

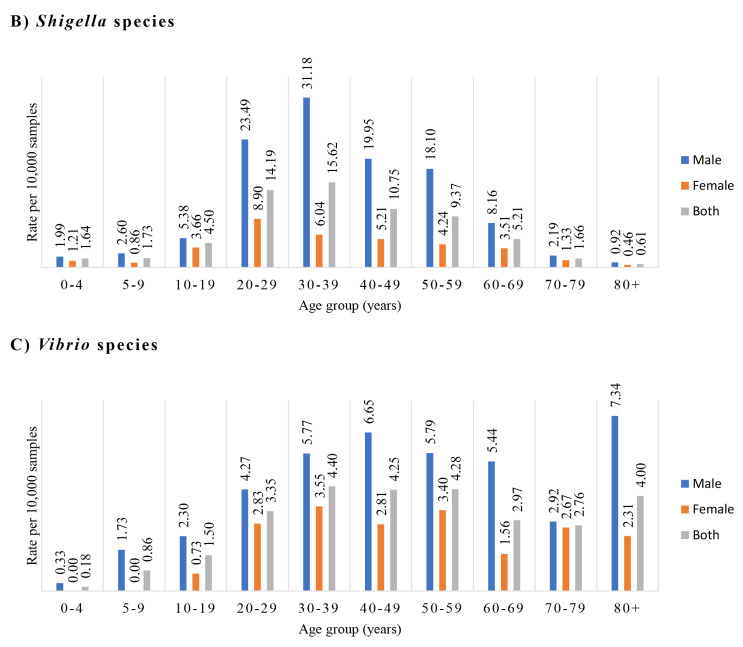
Isolation of *Plesiomonas* *shigelloides*, *Shigella* species and *Vibrio* species during 2015–2019 from different age groups and gender. The bacterial pathogens were isolated from faecal samples of patients with gastroenteritis. The isolation rates were the positive isolations per 10,000 samples. (**A**) *P. shigelloides*, (**B**) *Shigella* species and (**C**) *Vibrio* species. The isolations of *P. shigelloides*, *Shigella* and *Vibrio* species were more common during human adult life between 20 to 59 years old. The isolation rate of *Shigella* species was significantly higher in male patients than female patients, *p* < 0.001). The isolation rate of *Vibrio* species was positively associated with age (*p* < 0.001) and significantly higher in male patients than female patients (*p* < 0.001).

The isolations of *P. shigelloides*, *Shigella* and *Vibrio* species were more common in adults between 20 to 59 years old (Figure 2). The *P. shigelloides* isolation rate was not significantly associated with either gender or age, both had the *p*-values > 0.05. The isolation rate of *Shigella* species was not significantly associated with age, but significantly higher in male than female patients, *p* < 0.001 and odds ratio 2.964 (95% confidence interval, 2.289–3.87). The isolation rate of *Vibrio* species was significantly associated with increasing age, with *p* < 0.001 and odds ratio 1.014 (95% confidence interval, 1.007–1.021), it was also more often isolated from male than female patients, *p* < 0.001 and odds ratio 1.958 (95% confidence interval, 1.339–2.878). The positive isolations of *P. shigelloides, Shigella* and *Vibrio* species in individual years from 2015 to 2019 are shown in Table 4.

### 3.5. Isolations of Aeromonas, Salmonella and Vibrio Species Were Positively Associated with Increase of Temperature

**Figure 3:**
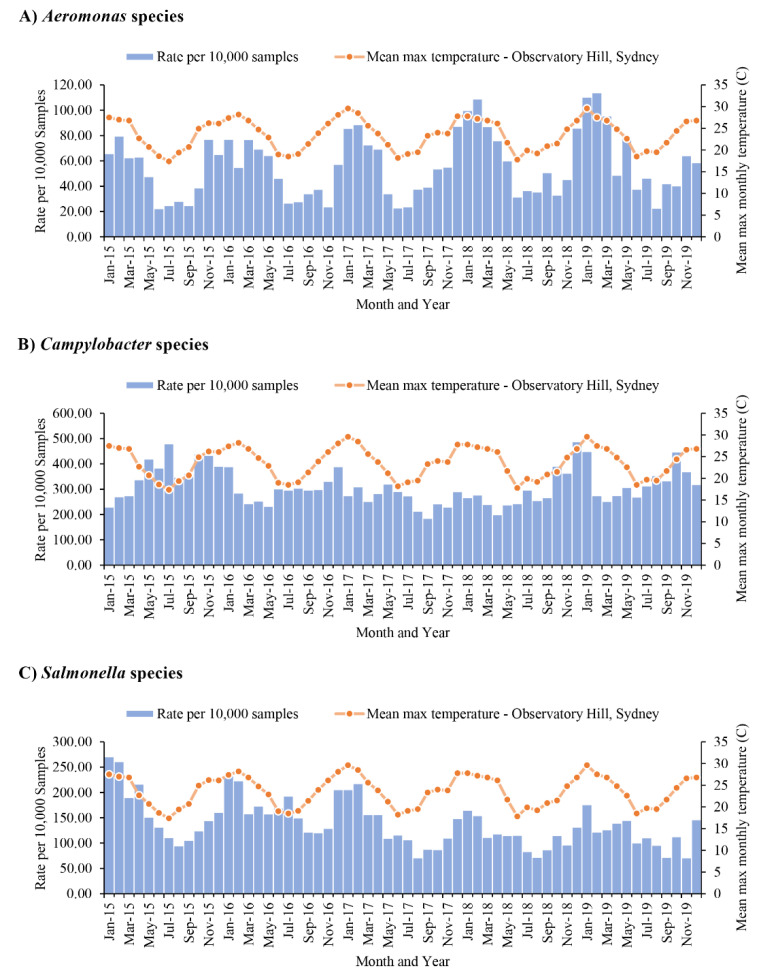
Isolation of *Aeromonas* species, *Campylobacter* species and *Salmonella* species in individual month during 2015–2019. The bacterial pathogens were isolated from faecal samples of patients with gastroenteritis. The isolation rates were the positive isolations per 10,000 samples. The mean maximum temperature was from Observatory Hill, Sydney. (**A**) *Aeromonas* species, (**B**) *Campylobacter* species and (**C**) *Salmonella* species. There was a strong positive correlation between *Aeromonas* species isolation rate and the local monthly mean maximum temperatures (*p* < 0.001, the Spearman’s rank correlation coefficient = 0.807). *Aeromonas* species were more often isolated in January, February, March and April each year. The isolation rates started to decrease in May and maintained at low isolation rates from June to November. In December, *Aeromonas* isolation started to increase again (Figure 3A). The isolation rate of *Campylobacter* species isolation was not significantly associated with the change of temperature, *p* = 0.835 and the Spearman’s rank correlation coefficient was −0.027 (Figure 3B). The isolation of *Salmonella* species was also positively associated with temperature, with *p* < 0.001 and the Spearman’s rank correlation coefficient 0.539 (Figure 3C).

There was a strong positive correlation between *Aeromonas* species isolation rate and the local monthly mean maximum temperatures (*p* < 0.001, the Spearman’s rank correlation coefficient = 0.807). *Aeromonas* species were more often isolated in January, February, March and April each year. The isolation rates started to decrease in May and maintained at low isolation rates from June to November. In December, *Aeromonas* isolation started to increase again, corresponding to the onset of the Australian summer (Figure 3A).

**Figure 4:**
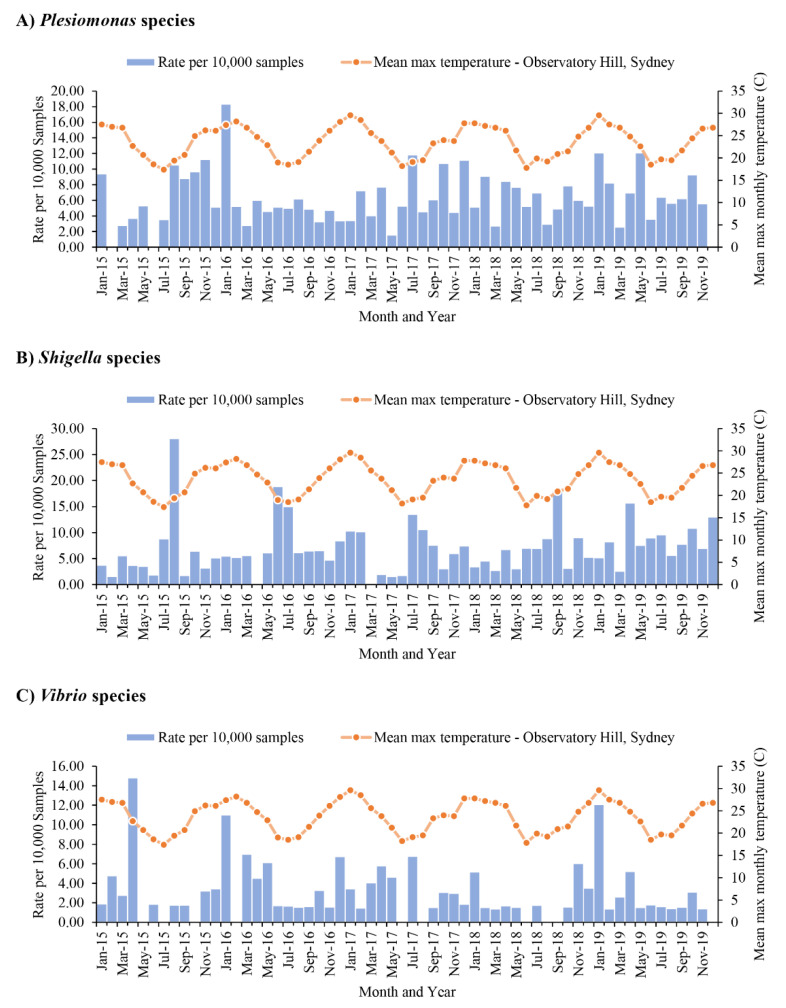
Isolation of *Plesiomonas* *shigelloides*, *Shigella* species and *Vibrio* species in individual month during 2015–2019. The bacterial pathogens were isolated from faecal samples of patients with gastroenteritis. The isolation rates were the positive isolations per 10,000 samples. The mean maximum temperature was from Observatory Hill, Sydney. (**A**) *P. shigelloides*, (**B**) *Shigella* species and (**C**) *Vibrio* species. There were no significant associations between the isolations of *P.* *shigelloides* and *Shigella* species with temperature. The isolation of *Vibrio* species was positively associated with temperature (*p* = 0.013).

The isolation rate of *Campylobacter* species was not significantly associated with the change of temperature, *p* = 0.835 and the Spearman’s rank correlation coefficient was −0.027 (Figure 3B). In contrast, the isolation of *Salmonella* species was also positively associated with temperature, with *p* < 0.001 and the Spearman’s rank correlation coefficient 0.539 (Figure 3C). There were no significant associations between the isolations of *P.* *shigelloides* and *Shigella* species with temperature. The isolation of *Vibrio* species showed a moderate positive correlation with temperature, *p* = 0.013 and the Spearman’s rank correlation coefficient 0.319 (Figure 4).

### 3.6. The Identities of Isolated Aeromonas Species

**Figure 5:**
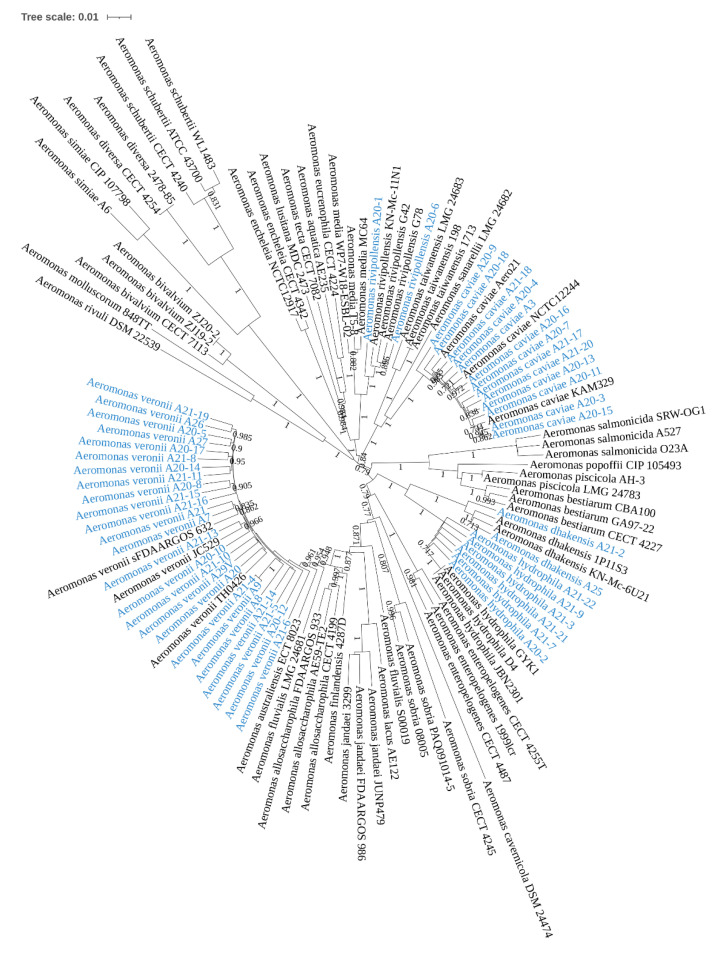
Identification of *Aeromonas* species through use of multilocus phylogenetic analysis. *Aeromonas* isolates were subjected to species identification based on the sequences of seven housekeeping genes, namely *gyr*B, *rpo*D, *gyr*A, *rec*A, *dna*J, *dna*X and *atp*D. *Bootstrap* values were generated from 1000 replicates. *Bootstrap* values between 0.7 and 1 are shown. Of the 48 *Aeromonas* isolates (blue) subjected to analysis, 25 isolates were *Aeromonas veronii*, 13 isolates were *Aeromonas caviae*, six isolates were *Aeromonas hydrophila*, two isolates were *Aeromonas dhakensis* and two isolates were *Aeromonas rivipollensis*.

A subset (48 isolates) of isolated *Aeromonas* species was identified to the species level, using MLPA. Five *Aeromonas* species were identified, of which 25 isolates were *Aeromonas veronii* (25%), 13 isolates were *Aeromonas caviae* (27%), six isolates were *Aeromonas hydrophila* (12.5%), two isolates were *Aeromonas dhakensis* (4.2%) and two isolates were *Aeromonas rivipollensis* (4.2%) (Figure 5).

## 4. Discussion

In this study, we examined the infection of *Aeromonas* species and an additional five enteric bacterial pathogens in human gastroenteritis by analysis of bacterial pathogen cultivation data from 375,842 stool samples processed in a large Australian diagnostic laboratory in five consecutive years, from 2015 to 2019. We also identified a subset of *Aeromonas* isolates to species level.

We found that *Aeromonas* species were the third most common bacterial pathogens in human gastroenteritis (Table 1). *Aeromonas* species were the only isolated bacterial pathogens in most patients with positive isolation, supporting their role in causing human gastroenteritis (Table 2). *Campylobacter* and *Salmonella* species were the top two most common bacterial pathogens causing human gastroenteritis, and most *Salmonella* species were non-typhoidal species (Table 1). Other enteric bacterial pathogens, such as *P.* *shigelloides*, *Shigella* and *Vibrio* species, were also isolated; the infections of these bacterial species in human gastroenteritis were much less common in comparison to *Campylobacter*, *Salmonella* and *Aeromonas* species (Table 1).

*Aeromonas* isolation rate was significantly correlated with increasing age. *Aeromonas* species’ isolation rate was low in young children, and it gradually increased as children got older. It reached the first small peak in the young-adult group of 20–29 years old and maintained a steady isolation rate in human adult life. Interestingly, the *Aeromonas* species isolation rate started to increase again in individuals over 50 years old and reached the second peak in the oldest age group age (more than 80 years old group) (Figure 1A and Table 3). This interesting isolation pattern was not seen in the other five bacterial pathogen species analysed in this study. *Campylobacter* and *Salmonella* species infections in human gastroenteritis were also age related, but their most commonly affected age groups were different from *Aeromonas* species infection. *Campylobacter* species more often affected individuals between 10 to 29 years old (Figure 1B), and *Salmonella* species most commonly affected young children (Figure 1C). The other three enteric pathogens, *P. shigelloides*, *Shigella* and *Vibrio* species, were mostly isolated from individuals of 20 to 59 years old (Figure 2 and Table 4). The novel infection pattern of *Aeromonas* in human gastroenteritis suggests that the two peaks are likely due to different reasons. The first peak most likely was due to increased exposure to the sources of infection. The continuous increase of *Aeromonas* infection in individuals after 50 years old, including the second peak, most likely was due to decreased host defence associated with human aging.

*Aeromonas* species infection detected in this study was predominantly locally acquired, with less than 5% of patients having a documented travel history. Previous studies have reported isolation of *Aeromonas* species from various sources, such as drinking water, fish, vegetables and freshwater sources, such as lakes and rivers [28]. The sources of *Aeromonas* species infection in Australian patients with gastroenteritis remain to be investigated. *Aeromonas* species associated gastroenteritis occurred more often in warm seasons, showing that the main infectious sources for human infections, once identified, should be particularly monitored for *Aeromonas* species load during warm seasons (Figure 3). The isolations of *Aeromonas*, *Campylobacter*, *Salmonella,* *Shigella* and *Vibrio* species were significantly higher from male patients than female patients, reflecting potentially a gender-based difference in lifestyle.

Five *Aeromonas* species were identified in our study for 48 *Aeromonas* isolates, and *A. veronii* was the most common species (52%), followed by *A. caviae* (27%) and *A. hydrophila* (12.5%) (Figure 5). In a recent study examining antibiotic resistance of *Aeromonas* species isolated from different infection sites, Sinclair et al. included 18 *Aeromonas* species isolated from stool samples in Queensland, Australia, of which 12 isolates (66.7%) were *A. veronii* and four isolates (22.2%) were *A. caviae* [29]. Our data and the study by Sinclair et al. consistently showed that *A. veronii* was the most common *Aeromonas* species isolated from stool samples of patients with gastroenteritis in the Australian population. In a review paper, Fernandez-Bravo et al. combined *Aeromonas* species isolated from faecal samples in different studies, showing that *A. caviae* had the highest isolation (49%), followed by *A. veronii* (25%), suggesting that the most common *Aeromonas* species isolated from stool samples in different regions vary [1]. Our study is also the first reporting *Aeromonas rivipollensis* isolation from human faecal samples. *A. rivipollensis* was previously isolated from river sediments [30].

In summary, this study found that *Aeromonas* species were the third most common enteric bacterial pathogen in Australian patients with gastroenteritis, following *Campylobacter* and *Salmonella* species. *Aeromonas* infection in patients with gastroenteritis was significantly correlated with increasing age, more often occurred in warm seasons and in male than female patients. *A. veronii* was the most common *Aeromonas* species isolated from patients with gastroenteritis in the Australian population. *Aeromonas* species infection detected in this study was predominantly locally acquired. In conclusion, the findings from this study support the importance of *Aeromonas* species in human gastroenteritis and suggest that the sources of *Aeromonas* infection in Australian patients should be further investigated.

## Figures and Tables

**Table 1:** Isolation of bacterial pathogens from faecal samples of patients with gastroenteritis in years 2015–2019 *.

	2015	2016	2017	2018	2019	Five-Year Total	Isolation Rate
*Campylobacter species*	2564	2282	1973	2201	2577	11597	308.56
*Salmonella species*	1155	1290	982	849	914	5190	138.09
*Salmonella* Paratyphi A	1	3	0	0	0	4	0.11
*Salmonella* Typhi	1	1	1	2	0	5	0.13
*Aeromonas species*	357	381	420	472	502	2132	56.73
*Plesiomonas shigelloides*	41	43	48	45	51	228	6.07
*Shigella species*	0	0	1	0	0	1	0.03
*Shigella boydii*	2	0	0	2	3	7	0.19
*Shigella dysenteriae*	1	0	0	0	1	2	0.05
*Shigella flexneri*	10	10	15	6	14	55	1.46
*Shigella sonnei*	29	45	30	41	47	192	5.11
*Vibrio species*	0	2	0	0	0	2	0.05
*Vibrio alginolyticus*	0	1	1	1	2	5	0.13
*Vibrio cholerae*	3	9	9	3	5	29	0.77
*Vibrio fluvialis*	0	1	0	0	1	2	0.05
*Vibrio parahaemolyticus*	18	16	12	11	14	71	1.89
*Yersinia enterocolitica*	4	5	3	6	11	29	0.77
*Yersinia enterocolitica group*	9	15	4	7	4	39	1.04
*Yersinia frederiksenii*	1	1	1	0	0	3	0.08
*Edwardsiella tarda*	2	5	4	0	5	16	0.43
*Comamonas testosteroni*	0	0	0	1	1	2	0.05
*Arcobacter species*	0	0	0	0	1	1	0.03
*Shewanella algae*	0	1	0	0	0	1	0.03

Detection of bacterial pathogens was through bacterial cultivation. * There were 70,613, 76,350, 75,253, 75,279 and 78,347 stool samples processed in the years of 2015, 2016, 2017, 2018 and 2019. Isolation rates refer to positive isolations per 10,000 faecal samples, which were calculated by using the bacterial pathogen isolations and the total samples numbers in five years (2015–2019).

**Table 2:** Isolation of *Aeromonas* species alone or co-isolation with other enteric bacterial pathogens in years 2015–2019 *.

	2015	2016	2017	2018	2019	Five-Year Total
*Aeromonas*	277	307	354	390	427	1755
*Aeromonas*, *Campylobacter*	49	45	48	68	59	269
*Aeromonas, Salmonella*	24	22	11	6	12	75
*Aeromonas, Plesiomonas*	3	2	3	5	2	15
*Aeromonas, Shigella*	1	1	1	0	0	3
*Aeromonas, Vibrio*	3	2	3	1	0	9
*Aeromonas, Yersinia*	0	2	0	2	2	6

* Numbers in the table are positive isolations.

**Table 3:** Isolation of *Aeromonas*, *Campylobacter* and *Salmonella* species in individual years, from 2015 to 2019, in different age groups.

	*Aeromonas* Positive Case		*Campylobacter* Positive Case		*Salmonella* Positive Case		Number of Stool Tested	
Age Group (Years)	Male	Female	Male	Female	Male	Female	Male	Female
2015								
0–4	8	11	173	109	126	122	6276	5075
5–9	11	3	101	56	48	60	2288	2285
10–19	26	5	192	87	64	65	2370	2463
20–29	15	20	211	210	57	82	2666	4681
30–39	18	24	148	179	75	84	3178	5316
40–49	23	20	123	120	45	48	2804	4675
50–59	14	22	125	136	37	59	2643	4488
60–69	15	25	136	140	28	60	2809	4861
70–79	15	22	115	103	24	20	2384	3751
80+	19	41	34	65	18	32	1812	3755
2016								
0–4	16	9	131	113	182	165	6404	5219
5–9	6	5	103	54	43	48	2381	2376
10–19	10	7	168	90	57	59	2509	2698
20–29	21	32	147	179	74	105	2775	5049
30–39	14	28	130	150	56	84	3664	5757
40–49	15	17	129	125	63	62	3007	5116
50–59	18	26	116	120	47	61	2750	4861
60–69	27	25	121	114	35	44	3013	5210
70–79	21	27	101	88	30	52	2691	4296
80+	24	33	47	52	7	18	2133	4379
2017								
0–4	13	18	118	87	128	113	5925	4990
5–9	11	7	70	42	35	27	2360	2344
10–19	14	9	133	71	60	47	2534	2622
20–29	18	24	127	143	44	68	2645	4862
30–39	15	28	139	123	43	57	3322	5488
40–49	15	30	88	105	37	44	2981	4996
50–59	20	27	101	91	42	66	2780	4720
60–69	17	30	107	150	31	53	2934	5073
70–79	27	41	95	93	24	36	2778	4715
80+	19	37	46	42	13	15	2409	4720
2018								
0–4	9	14	128	83	144	101	5619	4580
5–9	4	6	69	46	34	31	2224	2312
10–19	15	10	152	81	42	26	2770	2856
20–29	22	22	182	180	49	53	3014	5072
30–39	21	20	139	126	33	56	3533	5639
40–49	19	25	110	107	35	33	2994	5032
50–59	25	34	133	99	42	55	2775	4734
60–69	26	38	128	118	27	36	2862	5177
70–79	43	47	94	119	19	18	2883	4687
80+	20	52	37	68	7	12	2217	4234
2019								
0–4	10	12	133	89	137	102	5949	4960
5–9	12	4	86	60	49	43	2293	2318
10–19	11	7	179	108	42	39	2838	3020
20–29	32	26	207	190	37	76	2950	5048
30–39	25	31	160	167	42	50	3624	5942
40–49	21	33	140	124	33	50	3254	5134
50–59	20	31	143	150	29	39	2862	4755
60–69	35	38	146	138	39	42	3096	5310
70–79	26	55	131	104	26	23	2982	5059
80+	24	49	54	63	9	10	2326	4555

**Table 4:** Isolation of *Plesiomonas shigelloides*, *Shigella* and *Vibrio* species in individual years, from 2015 to 2019, in different age groups.

	*Plesiomonas* Positive Case		*Shigella* Positive Case		*Vibrio* Positive Case		Number of Stool Tested	
Age Group (Years)	Male	Female	Male	Female	Male	Female	Male	Female
2015								
0–4	0	0	1	2	0	0	6276	5075
5–9	0	0	1	0	1	0	2288	2285
10–19	2	1	0	0	0	0	2370	2463
20–29	4	1	2	5	1	0	2666	4681
30–39	2	4	3	3	3	2	3178	5316
40–49	4	2	8	2	2	1	2804	4675
50–59	3	7	8	3	2	1	2643	4488
60–69	3	4	2	2	1	0	2809	4861
70–79	2	0	0	0	0	1	2384	3751
80+	1	1	0	1	3	3	1812	3755
2016								
0–4	3	0	2	0	0	0	6404	5219
5–9	1	2	0	0	0	0	2381	2376
10–19	2	0	0	3	1	1	2509	2698
20–29	2	8	13	4	1	1	2775	5049
30–39	3	7	12	3	3	3	3664	5757
40–49	2	5	10	2	2	2	3007	5116
50–59	1	2	3	0	3	1	2750	4861
60–69	0	3	3	0	3	3	3013	5210
70–79	1	0	0	0	1	1	2691	4296
80+	0	1	0	0	2	1	2133	4379
2017								
0–4	0	1	1	1	1	0	5925	4990
5–9	0	0	1	1	0	0	2360	2344
10–19	1	2	4	0	1	0	2534	2622
20–29	2	2	4	4	1	3	2645	4862
30–39	1	4	8	3	3	2	3322	5488
40–49	7	4	5	4	2	2	2981	4996
50–59	6	5	4	2	0	2	2780	4720
60–69	2	3	2	1	2	0	2934	5073
70–79	4	2	0	1	0	1	2778	4715
80+	0	2	0	0	1	1	2409	4720
2018								
0–4	0	0	2	0	0	0	5619	4580
5–9	0	2	1	0	1	0	2224	2312
10–19	0	1	0	0	1	0	2770	2856
20–29	2	3	4	2	1	1	3014	5072
30–39	7	1	12	5	0	2	3533	5639
40–49	2	2	0	3	1	1	2994	5032
50–59	2	9	4	4	1	1	2775	4734
60–69	2	6	4	4	0	1	2862	5177
70–79	3	2	2	1	1	1	2883	4687
80+	0	1	1	0	2	0	2217	4234
2019								
0–4	0	2	0	0	0	0	5949	4960
5–9	0	1	0	0	0	0	2293	2318
10–19	2	0	3	2	0	0	2838	3020
20–29	5	8	10	7	2	2	2950	5048
30–39	3	6	19	3	1	1	3624	5942
40–49	2	4	7	2	3	1	3254	5134
50–59	3	4	6	1	2	3	2862	4755
60–69	4	3	1	2	2	0	3096	5310
70–79	1	1	1	1	2	2	2982	5059
80+	1	1	0	0	0	0	2326	4555

## Data Availability

Not applicable.
